# Characterization of a Functionally Unknown Arginine–Aspartate–Aspartate Family Protein From *Halobacillus andaensis* and Functional Analysis of Its Conserved Arginine/Aspartate Residues

**DOI:** 10.3389/fmicb.2018.00807

**Published:** 2018-04-25

**Authors:** Li Shao, Heba Abdel-Motaal, Jin Chen, Huiwen Chen, Tong Xu, Lin Meng, Zhenglai Zhang, Fankui Meng, Juquan Jiang

**Affiliations:** Department of Microbiology and Biotechnology, College of Life Sciences, Northeast Agricultural University, Harbin, China

**Keywords:** RDD family, moderate halophile, Na^+^(Li^+^, K^+^)/H^+^ antiporter, arginine/aspartate residues, site-directed mutagenesis

## Abstract

Arginine–aspartate–aspartate (RDD) family, representing a category of transmembrane proteins containing one highly conserved arginine and two highly conserved aspartates, has been functionally uncharacterized as yet. Here we present the characterization of a member of this family designated RDD from the moderate halophile *Halobacillus andaensis* NEAU-ST10-40^T^ and report for the first time that RDD should function as a novel Na^+^(Li^+^, K^+^)/H^+^ antiporter. It’s more interesting whether the highly conserved arginine/aspartate residues among the whole family or between RDD and its selected homologs are related to the protein function. Therefore, we analyzed their roles in the cation-transporting activity through site-directed mutagenesis and found that D154, R124, R129, and D158 are indispensable for Na^+^(Li^+^, K^+^)/H^+^ antiport activity whereas neither R35 nor D42 is involved in Na^+^(Li^+^, K^+^)/H^+^ antiport activity. As a dual representative of Na^+^(Li^+^, K^+^)/H^+^ antiporters and RDD family proteins, the characterization of RDD and the analysis of its important residues will positively contribute to the knowledge of the cation-transporting mechanisms of this novel antiporter and the roles of highly conserved arginine/aspartate residues in the functions of RDD family proteins.

## Introduction

In prokaryotes, Na^+^/H^+^ antiporters are ubiquitous transmembrane proteins that mainly catalyze the efflux of cytoplasmic Na^+^ and Li^+^ or sometimes also K^+^ driven by an electrochemical proton gradient generated by distinct transporters such as ion-pumping ATPases or respiratory complexes across cytoplasmic membranes (Padan and Schuldiner, 1994; Ito et al., 1999; Padan et al., 2005; Slonczewski et al., 2009; Quinn et al., 2012). This category of proteins were also designated Na^+^(Li^+^)/H^+^ antiporters due to the simultaneous existence of Na^+^/H^+^ and Li^+^/H^+^ antiport activity, some of which function sometimes as K^+^/H^+^ antiporters (Ito et al., 1999; Padan et al., 2005; Quinn et al., 2012). In *Escherichia coli*, two specific Na^+^/H^+^ antiporters, NhaA (Karpel et al., 1988) and NhaB (Pinner et al., 1992), and a non-specific Na^+^(Ca^2+^, K^+^)/H^+^ antiporter, ChaA (Ivey et al., 1993; Radchenko et al., 2006), were found to be essential for the growth of the host under high saline-alkaline stress. Therefore, *E. coli* mutants EP432 (Δ*nhaA*Δ*nhaB*; Padan et al., 2001), KNabc (Δ*nhaA*Δ*nhaB*Δ*chaA*; Nozaki et al., 1996), or TO114 (the different designation with the same genotype as KNabc; Radchenko et al., 2006), especially the latter two of which can’t tolerate 0.2 M NaCl or 5 mM LiCl, have been extensively used as a heterologous host to clone and express genes with Na^+^/H^+^ antiport activity. Due to the deficiency in one major K^+^/H^+^ antiporter, ChaA, *E. coli* KNabc may be also used for the identification of K^+^/H^+^ antiporters (Meng et al., 2014, 2017). Na^+^/H^+^ antiporters may be mainly classified into the monovalent Cation/Proton Antiporter 1 (CPA-1) family including NhaA (Karpel et al., 1988; Herz et al., 2003), NhaB (Pinner et al., 1992; Nakamura et al., 1996), NhaC (Ito et al., 1997), NhaD (Nozaki et al., 1998; Liu et al., 2005; Kurz et al., 2006; Zhang et al., 2014; Cui et al., 2016; Wang et al., 2017), NheE (Sousa et al., 2013), NhaP (Utsugi et al., 1998), NhaG (Gouda et al., 2001), and NhaH (Yang et al., 2006c), CPA-2 family (Saier et al., 1999) including NapA (Waser et al., 1992) and GerN (Southworth et al., 2001), and CPA-3 family including six-or-seven-gene monovalent cation/proton antiporters such as *mrp* (Meng et al., 2014; Hamamoto et al., 1994; Dzioba-Winogrodzki et al., 2009; Cheng et al., 2016), *mnh* (Hiramatsu et al., 1998), *pha* (Putnoky et al., 1998; Jiang et al., 2004; Yang et al., 2006a; Yamaguchi et al., 2009) or *sha* (Kosono et al., 1999). Besides the above-mentioned CPA1-3 families, some proteins in other families have also been continually reported to possess Na^+^/H^+^ or Na^+^(K^+^)/H^+^ antiport activity, which include MF (major facilitator) family multi-drug transporters, MdfA (Lewinson et al., 2004), MdtM (Holdsworth and Law, 2013) and TetA(L) (Cheng et al., 1994), and HCT (2-hydroxy-carboxylate transporter) family transporter, MleN (Wei et al., 2000), and NDH (NADH dehydrogenase) family primary Na^+^ pump, Nap (Yang et al., 2006b), and PSMR (paired small multidrug resistance) family protein pair, PsmrAB (Jiang et al., 2013a).

Most bacteria contain 5–9 genes and operons encoding distinct putative Na^+^/H^+^ antiporters (Krulwich et al., 2009; Mesbah et al., 2009). In contrast, we speculate that the halophilic bacteria isolated from high saline-alkaline conditions may have evolved higher numbers of Na^+^/H^+^ antiporters including even novel ones that have not been reported as yet. That has been partially established by the recent report by Meng et al. (2017) that a pair of functionally unknown homologous DUF1538 family proteins from the moderate halophile *Halomonas zhaodongensis* function as a novel two-component Na^+^(Li^+^, K^+^)/H^+^ antiporter. As the type strain of a novel species of *Halobacillus andaensis* (Wang et al., 2015), NEAU-ST10-40^T^ is a moderate halophile isolated from unique Na_2_CO_3_-type saline and alkaline conditions, which can grow at the range of NaCl concentrations of 0.5–2.5 M (optimum, 1.4 M) and pH 7.0–9.0 (optimum, pH 8.0). Therefore, this novel halophile may have evolved profound mechanisms for the stability of its intracellular osmotic and ionic state. Since Na^+^/H^+^ antiporters are employed by almost all halophiles to extrude excessive Na^+^ in the cells (Ventosa et al., 1998; Oren, 1999), we speculate that this novel strain NEAU-ST10-40^T^, a moderate halophile which can tolerate up to 2.5 M NaCl, may contain many important (even novel) Na^+^/H^+^ antiporter genes.

For cloning novel Na^+^/H^+^ antiporter genes, genomic DNA was screened from *H. andaensis* NEAU-ST10-40^T^ by functional complementation with *E. coli* KNabc. All screened resultant genes have not been reported to possess Na^+^/H^+^ antiport activity as yet. For example, a UPF0118 family protein with uncharacterized function from this strain has recently been reported to represent a novel class of Na^+^(Li^+^)/H^+^ antiporters (Dong et al., 2017). Of other genes, one gene designated *rdd* showed the highest identity of 64% with an unannotated gene encoding an uncharacterized protein belonging to arginine–aspartate–aspartate (RDD) family from *Pontibacillus halophilus*, but shares no identity with all known genes with Na^+^/H^+^ antiport activity. In this study, we reported the cloning and characterization of *rdd* and propose that RDD should represent a novel class of Na^+^(Li^+^, K^+^)/H^+^ antiporters. More importantly, considering the conservation of six arginine/aspartate residues among the whole RDD family or between RDD and its selected homologs and the functional importance of charged arginine and aspartate residues for some identified Na^+^/H^+^ antiporters (Inoue et al., 1995; Noumi et al., 1997; Hellmer et al., 2003; Habibian et al., 2005; Hunte et al., 2005; Jiang et al., 2013b; Li et al., 2014), we used site-directed mutagenesis to analyze the relationship between conserved arginine/aspartate residues and Na^+^(Li^+^, K^+^)/H^+^ antiport activity of RDD.

## Materials and Methods

### Strains, Plasmids, and Growth Conditions

The strains and plasmids related to this study are shown in **Table 1**. *H. andaensis* NEAU-ST10-40^T^ was cultured in a modified S–G liquid medium with the composition as described by Wang et al. (2015). *E. coli* strain KNabc (Δ*nhaA*Δ*nhaB*Δ*chaA*; Nozaki et al., 1996) and its transformants were cultured in the LBK medium composed of 1.0% tryptone, 0.5% yeast extract, and 87 mM KCl as described by Karpel et al. (1988) or KCl-removed LBO (“O” stands for 0 mM KCl) medium solely consisting of 1.0% tryptone and 0.5% yeast extract. *E. coli* KNabc transformants were pre-cultured in the LBK or LBO media at pH 7.0, followed by incubation to the optical density of 1.0 at 600 nm (OD_600 nm_ = 1.0) at 37°C. As a preliminary growth test, 1% pre-cultures of *E. coli* KNabc transformant cells carrying the recombinant plasmid pUC-SL38 or pUC18 (a negative control) were inoculated into fresh LBK medium at pH 7.0 and the growth curves were plotted on a semilogarithmic scale. For the salt tolerance test, 1% pre-cultures were inoculated into fresh LBK media supplemented by NaCl, LiCl or KCl as indicated concentrations at pH 7.0. For the growth test under alkaline pH, 1% pre-cultures were inoculated into fresh LBK media with or without the addition of 50 mM NaCl, or KCl-removed LBO media at indicated pHs adjusted by a final concentration of 100 mM Hepes–Tris buffer. 50 mM NaCl was supplemented to the medium for growth tests under alkaline pH, because Na^+^(Li^+^)/H^+^ antiporters without K^+^/H^+^ antiport activity can function only in the presence of Na^+^ or Li^+^ (Padan and Schuldiner, 1994; Ito et al., 1999; Padan et al., 2005). However, to test the function of RDD as a K^+^/H^+^ antiporter, the growth tests were carried out in the LBK medium or even in the LBO medium without the addition of KCl. Also, NhaD (Wang et al., 2017) from *Halomonas alkaliphila* was introduced as a positive control of the sole Na^+^(Li^+^)/H^+^ antiporter without K^+^/H^+^ antiport activity whereas UmpAB (Meng et al., 2017) from *H. zhaodongensis* was introduced as a positive control of an Na^+^(Li^+^, K^+^)/H^+^ antiporter. The above-mentioned growth tests were carried out by evaluating the values for OD_600 nm_ after 24-h incubation at 37°C. Ampicillin or kanamycin was used for the selection and growth of *E. coli* KNabc transformants at a final concentration of 50 μg.ml^-1^. Preparation and electroporation of electrocompetent *E. coli* cells were carried out as described by Dong et al. (2017).

**Table 1:** Bacterial strains and plasmids used in this study.

Strains or plasmids	Relevant phenotype or genotype	The source
**Strains**		
*Halobacillus andaensis* NEAU-ST10-40^T^	Type strain of *Halobacillus andaensis*, a moderate halophile	Isolated and identified by our group (Wang et al., 2015)
*Escherichia coli* KNabc	Δ*nhaA*Δ*nhaB*Δ*chaA*	Donated by Professor Terry A. Krulwich (Nozaki et al., 1996)
**Plasmids**		
pUC18	Cloning vector	Takara Biotechnology (Dalian) Co. Ltd., China
pUC-SL38	pUC18 carrying a 1145-bp DNA fragment with Na^+^/H^+^ antiport activity	This study
pET19b	Over-expression vector, Amp^R^	Novagen Ltd., United States
pET19b-truncated SppA	pET19 carrying 5′-end truncated SppA	This study
pET22b	Over-expression vector, Amp^R^	Novagen Ltd., United States
pET28b	Over-expression vector, Kan^R^	Novagen Ltd., United States
pET28b-RDD	pET28b carrying the *rdd* gene fused in frame with an N-terminal His_6_ tag followed by a thrombin proteolytic cleavage site and a T7 tag	This study
pET22b-RDD	pET22b carrying the *rdd* gene fused in frame with an N-terminal His_6_ tag preceded by a T7 promoter	This study
pET22b-PRO-RDD	pET22b carrying the *rdd* gene fused in frame with an N-terminal His_6_ tag preceded by its native promoter instead of the T7 promoter	This study


### Screening and Subcloning of the Gene With Na^+^/H^+^ Antiport Activity

Screening of the gene with Na^+^/H^+^ antiport activity from strain NEAU-ST10-40^T^ was performed through a series of routine procedures for DNA manipulation and functional complementation with *E. coli* KNabc on the LBK medium plates containing 0.2 M NaCl, as described in the study by Dong et al. (2017). A tiny modification is that DNA fragments with both 4–10 kb and 0–10 kb were used for the ligation with pUC18 to increase the possibility for obtaining genes with Na^+^/H^+^ antiport activity. Although DNA fragments solely with 4–10 kb are screened in the routine protocols, we speculate that it’s very likely to miss the target genes possibly solely included in DNA fragments below 4 kb but not in those between 4 kb and 10 kb due to random partial digestion of genomic DNA. That was established by the following result that a 1145-bp DNA fragment was obtained in this study. Either ORF included in the 1145-bp DNA fragment inserted in the recombinant plasmid pUC-SL38 was subcloned separately to analyze which one can complement functionally with *E. coli* KNabc. The subcloning strategy for *rdd* gene was carried out similarly to that of *upf0118* gene, as described by Dong et al. (2017). To reflect the response of *rdd* gene to the change of saline-alkaline stress, its predicted native promoter and ribosomal site (RBS) was inserted between the corresponding *Bgl*II and *Nco*I sites of pET22b (Novagen Ltd., United States) to replace DNA fragment including the T7 promoter and RBS between the two enzymatic sites of the expression vector through PCR amplification, purification, double digestion, and ligation. The subcloning of 5′-end truncated *sppA* was performed by being fused with an N-terminal His_6_ tag in an expression vector pET19b (Novagen Ltd., United States). The sequences of primers are shown in Supplementary Table S1. All the resultant constructs including pET28b-RDD, pET22b-RDD, pET22b-PRO-RDD, and pET19b-truncated SppA (**Table 1**) were verified by sequencing analysis.

### Site-Directed Mutagenesis of Wild-Type RDD

For the construction of RDD variants, site-directed mutagenesis was carried out using pET22b-PRO-RDD as the template via a Fast Mutagenesis System kit purchased from TransGen Biotech Co., Ltd. (Beijing, China) including a TransStart^®^FastPfu DNA Polymerase with the high extension rate and high fidelity and a DMT enzyme used for the digestion of parental plasmids. Each pair of partially overlapping primers containing the target mutagenic sites were designed according to the above-mentioned kit instructions and synthesized by Beijing Genomics Institute (Beijing, China), as listed in Supplementary Table S1. The accuracy of final RDD variants in pET22b-PRO-RDD were confirmed by re-sequencing, and each of the corresponding plasmids was individually transformed to *E. coli* KNabc competent cells for growth tests and Na^+^(Li^+^, K^+^)/H^+^ antiport assay.

### Preparation of Cell Extract, Membrane Fraction (Everted Membrane Vesicles) and Cytoplasmic Fraction

Everted membrane vesicles were prepared for the following Na^+^(Li^+^, K^+^)/H^+^ antiport assay according to the previous method as described by Rosen (1986). The detailed protocol was shown as follows. *E. coli* KNabc cells carrying pET22b-PRO-RDD or its variants, or the empty vector pET22b (as a negative control), were grown in the LBK medium to the same OD_600 nm_ around 1.0 and harvested by centrifugation at 5000 *g*, 4°C for 10 min. The harvested cells were washed three times with a buffer containing 10 mM Tris–HCl (pH 7.5), 140 mM choline chloride, 0.5 mM dithiothreitol (DTT), and 250 mM sucrose, a protease inhibitor tablet (Roche), 1 mM phenylmethylsulfonyl fluoride (PMSF), and then lysed at 2,000 psi by using a JG-1A French pressure cell press (NingBo Scientz Biotechnology Co., Ltd., China). The cell debris and unlysed cells were removed from cell lysate through the centrifugation at 5,000*g*, 4°C for 10 min. The resulting supernatant including membrane and cytoplasmic fractions was partially sampled as a representative of cell extract and the remaining one continued to go through 1-h ultracentrifugation at 100,000 *g*. Membrane fraction (pellets) were finally separated from cytoplasmic fraction (supernatant) and re-suspended in the same above-mentioned buffer. It should be stressed that cytoplasmic membranes lysed and prepared by using the above-mentioned method exist as inside-out vesicles and therefore designated everted membrane vesicles. A small amount of cytoplasmic fraction was also sampled and re-suspended in the same volume of the above-mentioned buffer as before ultracentrifugation. All the above-mentioned prepared samples were stored at -80°C before use.

### SDS-PAGE and Western Blot

SDS-PAGE and western blots were carried out according to the routine protocol described by Green and Sambrook (2012). For the localization of RDD in the cytoplasmic membranes, cell extract, membrane fraction, and cytoplasmic fraction from *E. coli* KNabc/pET22b-PRO-RDD or KNabc/pET22b were analyzed by SDS-PAGE and western blots. To test the expression levels of wild-type RDD or its variants, the samples as the respective representatives of membrane fractions were guaranteed to be prepared from the same volume of *E. coli* KNabc cell cultures carrying pET22b-PRO-RDD (as a positive control) or its variants grown to the same OD_600 nm_ around 1.0 and the loaded amounts equivalent to 50 μg of total membrane protein were subjected to western blot analysis. A mouse anti-His_6_-tag antibody (Beyotime Biotechnology Co., Ltd., China) and a goat anti-mouse secondary antibody conjugated with a horseradish peroxidase (HRP; Beyotime Biotechnology Co., Ltd., China) were employed for the detection of RDD or its variants fused with His_6_ tag. The chemiluminescence detection was performed using Luminata crescendo western HRP substrate (Nachuan Biotechnology Co., Ltd., Changchun, China) via a Tanon 5200 Multi chemiluminescence imaging system (Tanon Co. Ltd., China).

### Na^+^(Li^+^, K^+^)/H^+^ Antiport Assay

A acridine orange fluorescence dequenching method was employed for the measurement of Na^+^(Li^+^, K^+^)/H^+^ antiport activity on the basis of everted membrane vesicles, as described by Rosen (1986). The vesicles (equivalent of 20 μg total membrane protein) were re-suspended in the assay mixture containing 140 mM choline chloride, 5 mM Mg_2_SO_4_, 1 μM acridine orange at the indicated pH values from 6.5 to 9.5 adjusted by a 10 mM BTP (Bis-Tris Propane) buffer. The fluorescence quenching with the acridine orange as a pH indicator was initiated by the addition of Tris–D-lactic acid at the final concentration of 10 mM, due to the respiration-coupled proton translocation from the outside of the vesicles into the inside. When the fluorescence quenching reached the steady state, a respiration-dependent proton gradient across the vesicles was constructed. After NaCl, LiCl or Na-free KCl was added to the final concentration of 10 mM, the fluorescence dequenching could be caused by the influx of Na^+^, Li^+^, or K^+^ into the vesicles in exchange for proton efflux. Na-free KCl with the purity of 99.9995% (Sigma-Aldrich Co., LLC) was used in this study to avoid the interference by traces of contaminated NaCl. Na^+^(Li^+^, K^+^)/H^+^ antiport activity could be finally represented, respectively, by the ratio of fluorescence dequenching extent by the corresponding cations to the fluorescence quenching one by Tris–D-lactic acid. Fluorescence was monitored at excitation and emission wavelength of 492 and 526 nm, respectively, with a Hitachi F-7000 fluorescence spectrophotometer (Hitachi Ltd., Tokyo, Japan).

### Calculation of K_0.5_ Values of RDD or Its Variants for the Transported Cations

For the analysis of the apparent affinity of RDD or its variants for the transported cations, K_0.5_ values were calculated as the representatives of half-maximal cation concentrations for Na^+^(Li^+^, K^+^)/H^+^ antiport activity. For this purpose, the optimal Na^+^(Li^+^, K^+^)/H^+^ antiport activities were measured, respectively, by varying the corresponding cation concentrations from 0 to 20 mM at pH 9.0. K_0.5_ values of RDD or its variants for Na^+^, Li^+^, and K^+^ were finally obtained, respectively, through the non-linear regression analysis of the plotting with the antiport activities as functions of their corresponding cation concentrations by Prism 5.0.

### DNA Manipulation and Sequence Analysis

DNA manipulation related to this study was carried out according the routine protocols described by Green and Sambrook (2012). DNA was sequenced by Beijing Genomics Institute (Beijing, China). ORF and hydrophobicity were analyzed using the software DNAMAN 6.0. Protein sequence was aligned using BlastP at the NCBI (National Center for Biotechnology Information) website https://blast.ncbi.nlm.nih.gov/Blast.cgi?PROGRAM=blastp&PAGE_TYPE=BlastSearch&LINK_LOC=blasthome (Altschul et al., 1990). The multiple alignments of RDD and its selected homologs, as well as all known single-gene proteins with the Na^+^/H^+^ antiport activity were performed by ClustalX program (Thompson et al., 1997). A neighbor-joining phylogenetic tree was constructed (Saitou and Nei, 1987), followed by a bootstrap analysis (1000 replications) for the stability of clusters. Promoter sequence was predicted at the website http://www.fruitfly.org/seq_tools/promoter.html (Reese, 2001). Transmembrane segments (TMSs) were predicted at the website http://www.tcdb.org/progs/TMS.php (Saier et al., 2016). An amino acid sequence logo was carried out by using the website http://weblogo.berkeley.edu/ (Crooks et al., 2004).

### Protein Content Determination

Protein concentration was analyzed using bovine serum albumin as a standard according to the method described by Lowry et al. (1951).

### Nucleotide Sequence Accession Number

The nucleotide sequence related to this study has been deposited with the accession number KY575967 to GenBank database.

## Results

### Cloning and Sequence Analysis of the Gene With Na^+^/H^+^ Antiport Activity

**FIGURE 1:**
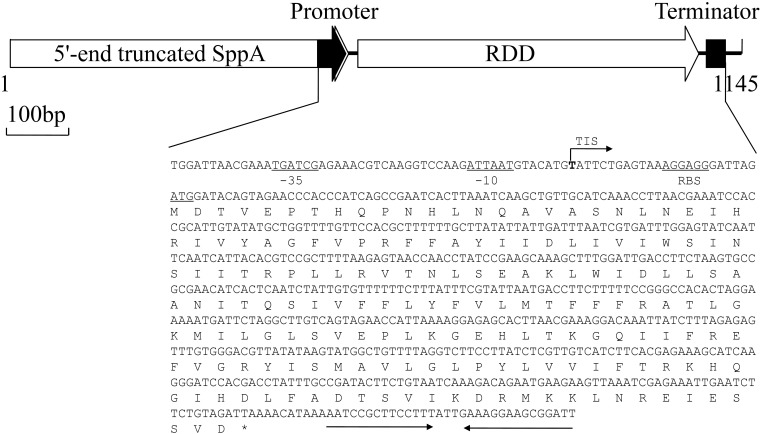
Mapping of the inserted DNA fragment in the recombinant plasmid pUC-SL38. One 5′-end truncated ORF1 designated SppA and one intact ORF2 designated RDD are included in a 1145-bp DNA fragment (GenBank accession No. KY575967) inserted into the recombinant plasmid pUC-SL38, the latter of which is preceded by a respective promoter-like sequence and a respective Shine–Dalgarno (SD) sequence. The predicted promoter sequence (-35 region and -10 region), the SD sequence, the ribosomal binding site (RBS) and the initiation codon ATG of RDD are underlined. The transcription initiation site (TIS) is highlighted in bold and also marked with the leftward arrow. The stop codon TAA of ORF2 is indicated by the asterisk and a possible terminator following RDD indicated by the inverted solid arrows.

**FIGURE 2:**
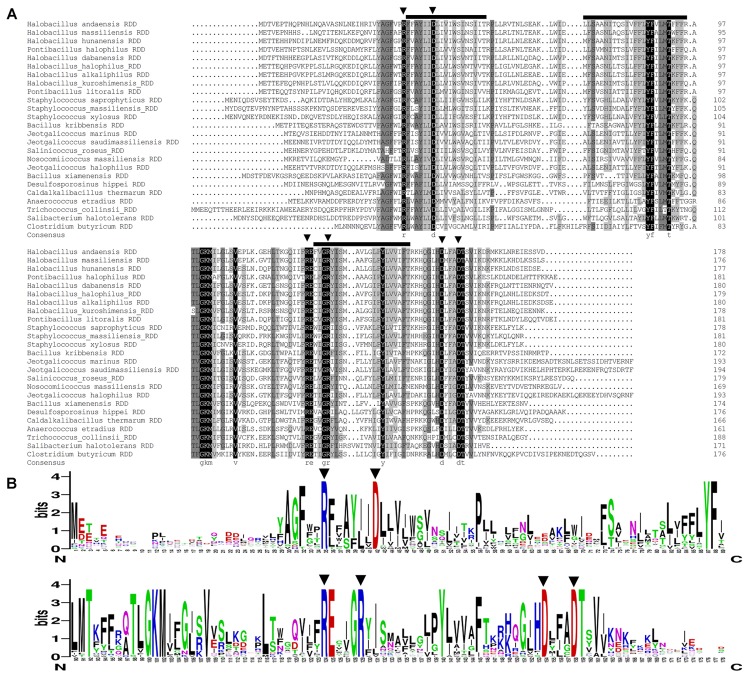
Alignment of RDD with all its selected holomogs and its web-based amino acid sequence logo. **(A)** Alignment of RDD with all its selected holomogs. RDD was aligned with all its selected homologs with 26–74% identities within the neighbor-joining phylogenetic tree (**Figure 9**) to show its conserved amino acid residues. Accession.version numbers and hosts of the selected homologs are shown in the neighbor-joining phylogenetic tree of **Figure 9**. Shading homology corresponds to 100% (black), ≥75% (gray), ≥50% (light gray), and <50% (white) amino acid identity, respectively. The three putative transmembrane segments are marked with bold lines above the alignment. **(B)** RDD sequence logo. An amino acid sequence logo was also created by submitting the multiple sequence alignment of RDD with the above-mentioned 24 homologs to the website http://weblogo.berkeley.edu/. The heights of amino acid symbols stand for their conservation in the multiple alignment. The highly conserved arginine (R) and aspartate (D) residues between RDD and all its selected homologs with 26–74% identities are highlighted with the downward filled triangles above the alignment (A) or sequence logo (B).

For the cloning of Na^+^/H^+^ antiporter genes, genomic DNA from strain NEAU-ST10-40^T^ was partially digested by *Sau*3AI and its ligation mixture with a cloning vector pUC18 was electroporated into *E. coli* KNabc, followed by the screening of its transformants by functional complementation on the LBK medium plates containing 0.2 M NaCl. As a result, one recombinant plasmid designated pUC-SL38 was found to succeed in complementing with *E. coli* KNabc. On the basis of sequence analysis, a 1145-bp DNA fragment was found to contain one 5′-end truncated open reading frame (ORF1) and one intact ORF2 (**Figure 1**), both of which were inserted in the opposite orientation just downstream from the *lac* promoter of pUC18. The sole ORF2, but not the 5′-end truncated ORF1, is preceded by a promoter-like sequence and a SD sequence (**Figure 1**). The 5′-end truncated ORF1 shares the highest identity of 81.8% with the amino acid sequence from No. 155 residue to No. 329 residue of a putative signal peptide peptidase A consisting of 329 residues (SppA, accession version No. WP_082235112.1) from *Halobacillus massiliensis*, and ORF2 shares the highest identity of 73.6% with an RDD family protein (accession.version No. WP_082235113.1, the corresponding gene is unannotated in its genome) from *H. massiliensis* (**Figure 2A**). Also, ORF2 shares the higher identities of 66.7%, 64.6%, 62.9%, 58.4%, 57.9%, 57.3%, and 56.7% with RDD family proteins from *Halobacillus hunanensis*, *Pontibacillus yanchengensis*, *Halobacillus dabanensis*, *Halobacillus halophilus*, *Halobacillus alkaliphilus*, *Halobacillus kuroshimensis*, and *Pontibacillus litoralis* (**Figure 2A**).

The above-mentioned two ORFs were aligned, respectively, with all known specific Na^+^/H^+^ antiporters and proteins with Na^+^/H^+^ antiport activity, even putative Na^+^/H^+^ antiporters, but either one showed no identity with each of them. However, ORF2, an RDD family protein with uncharacterized function, is predicted to be the sole transmembrane protein consisting of three putative TMSs including TMS I (36–54), TMS II (72–96), and TMS III (126–146) (**Figure 2A**). The molecular weight and pI of ORF2 were calculated to be 20, 419.5 Dalton and 9.72, respectively. More importantly, ORF2 is found to be composed of 178 amino acid residues, of which 105 residues were hydrophobic, and ORF2 is therefore of low polarity. Considering Na^+^/H^+^ antiporters must be transmembrane proteins of low polarity (Padan and Schuldiner, 1994; Ito et al., 1999; Padan et al., 2005; Quinn et al., 2012), we speculate that ORF2 may possess Na^+^/H^+^ antiport activity and designate this gene *rdd* for the description related to its following identification.

### Identification of ORF With Na^+^/H^+^ Antiport Activity

**FIGURE 3:**
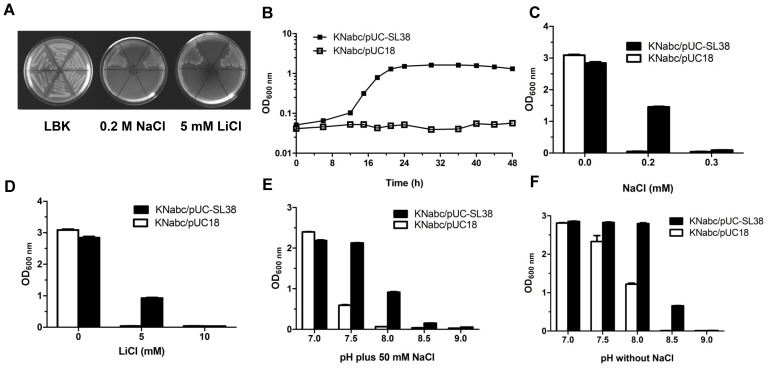
Growth tests for *E. coli* KNabc transformants under saline or alkaline conditions. For the complementation test **(A)**, *E. coli* strains KNabc transformant cells were grown on the LBK medium plates at pH 7.0 containing no addition of NaCl or LiCl, 0.2 M NaCl or 5 mM LiCl. (1) KNabc/pUC-SL38, (2) KNabc/pUC18, (3) KNabc/pET22b-PRO-RDD, (4) KNabc/pET22b, (5) KNabc/pET19b-truncated SppA, (6) KNabc/pET19b. As a preliminary growth test **(B)**, the growth curves of *E. coli* KNabc/pUC-SL38 and KNabc/pUC18 in the presence of 0.2 M NaCl were plotted on a semilogarithmic scale. For the growth tests under saline or alkaline conditions, *E. coli* KNabc/pUC-SL38 and KNabc/pUC18 were grown in the LBK media containing 0–0.3 M NaCl **(C)** or 0–10 mM LiCl **(D)**, or with **(E)** or without **(F)** the addition of 50 mM NaCl at the pH values from 7.0 to 9.0. The pre-cultures of *E. coli* KNabc transformant cells were grown to OD_600 nm_ at 1.0 in the LBK medium at pH 7.0 at 37°C. The above-mentioned cell growth was ended after 24 h and the values for OD_600 nm_ then evaluated. Each data point represents the average of three independent determinations.

For the identification of the exact ORF with Na^+^/H^+^ antiport activity, *rdd* gene was constructed as the priority into an expression vector pET22b through the fusion of the sole ORF of RDD in frame with an N-terminal His_6_ tag and the resultant construct was designated pET22b-RDD (**Table 1**). To reflect the response of *rdd* gene to the change of saline-alkaline stress, the T7 promoter of pET22b was further replaced by the predicted native promoter of *rdd* gene and the resultant construct was designated pET22b-PRO-RDD (**Table 1**). Although 5′-end truncated ORF1 without the aid of promoter or SD sequences can’t be theoretically transcribed or translated in *E. coli*, it was also subcloned by being fused with an N-terminal His_6_ tag of pET19b and the resultant construct was designated pET19b-truncated SppA (**Table 1**). Either subclone was tested by functional complementation of its transformant with *E. coli* KNabc. As shown in **Figure 3A**, all *E. coli* KNabc transformants showed the normal growth in the absence of NaCl or LiCl. In contrast, *E. coli* KNabc/pET22b-PRO-RDD exhibited the similar growth with KNabc/pUC-SL38 on the LBK medium plates containing 0.2 M NaCl or 5 mM LiCl whereas the negative controls KNabc/pET22b, KNabc/pUC18, or KNabc/pET19b, as well as KNabc/pET19b-truncated SppA, showed no growth under the same stress conditions (**Figure 3A**). Therefore, RDD is exactly likely to function as a Na^+^/H^+^ antiporter, since Na^+^/H^+^ antiporters can catalyze the efflux of cytoplasmic Na^+^ and Li^+^ (Padan and Schuldiner, 1994; Ito et al., 1999; Padan et al., 2005).

### Resistance of RDD to the Salts and Alkaline pH

Na^+^/H^+^ antiporters can extrude cytoplasmic Na^+^ and Li^+^ by exchanging with external protons (Padan and Schuldiner, 1994; Ito et al., 1999; Padan et al., 2005). Therefore, Na^+^(Li^+^)/H^+^ antiporters or proteins with the sole Na^+^(Li^+^)/H^+^ antiport activity must possess the ability of the tolerance to NaCl or LiCl and also exhibit the Na^+^/Li^+^-dependent alkaline pH resistance. As a preliminary growth test, the growth curves of *E. coli* KNabc/pUC-SL38 and KNabc/pUC18 were plotted as the priority on a semilogarithmic scale. The growth of *E. coli* KNabc/pUC-SL38 was found to increase significantly after 12 h and reach the stationary phase at 24 h in the presence of 0.2 M NaCl whereas KNabc/pUC18 could not grow under the same stress conditions (**Figure 3B**). Therefore, the following growth tests were ended on the time point of 24 h. Further salt tolerance test showed that *E. coli* KNabc/pUC-SL38 could grow in the presence of 0.2 M NaCl or 5 mM LiCl, but not *E. coli* KNabc/pUC18 (**Figures 3C,D**). Alkaline pH resistance test in the presence of 50 mM NaCl showed that the growth of *E. coli* KNabc/pUC18 was significantly inhibited as pH increased from the neutral to the alkaline, in contrast, the expression of *rdd* conferred alkaline pH resistance to *E. coli* KNabc (**Figure 3E**).

**FIGURE 4:**
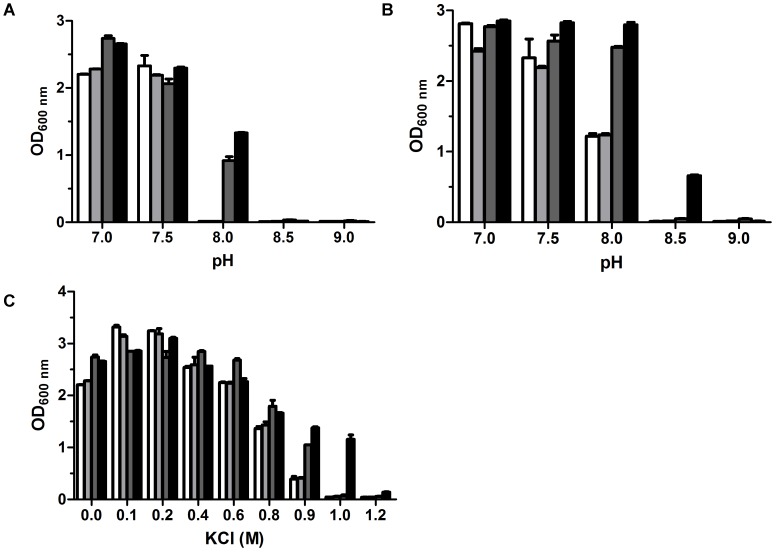
Growth tests for the function of RDD as a K^+^/H^+^ antiporter. To test whether RDD is likely to function as a K^+^/H^+^ antiporter, *E. coli* KNabc/pUC-SL38 (black column), KNabc/pUC-umpAB (gray column) as a positive control of a Na^+^(Li^+^, K^+^)/H^+^ antiporter (Meng et al., 2017) and KNabc/pUC-nhaD (light gray column) as a positive control of the sole Na^+^(Li^+^)/H^+^ antiporter (Wang et al., 2017) and KNabc/pUC18 (white column) as a negative control were grown in the KCl-removed LBO media **(A)** or LBK media **(B)** at the pH values from 7.0 to 9.0. Furthermore, the above-mentioned *E. coli* KNabc transformants were also grown in LBK media containing 0–1.2 M KCl **(C)**. The pre-cultures of *E. coli* KNabc transformant cells was grown to OD_600 nm_ at 1.0 in the LBO medium at pH 7.0 at 37°C. The above-mentioned cell growth was ended after 24 h and the values for OD_600 nm_ then evaluated. Each data point represents the average of three independent determinations.

It should be stressed that the expression of *rdd* conferred *E. coli* KNabc cells a significantly higher resistance to alkaline pH at 8.0 and even 8.5 in the LBK media without the addition of NaCl (**Figure 3F**) than in the same media with the addition of 50 mM NaCl (**Figure 3E**). We speculate that the presence of K^+^ in the LBK media may lead to this difference. Since Na^+^/H^+^ antiporters have been reported to sometimes possess K^+^/H^+^ antiport activity to support K^+^-dependent intracellular pH homeostasis under alkaline conditions (Putnoky et al., 1998; Ito et al., 1999; Lewinson et al., 2004; Padan et al., 2005; Quinn et al., 2012; Holdsworth and Law, 2013; Cheng et al., 2016), it’s very likely that RDD may also function as a K^+^/H^+^ antiporter and it can therefore employ limited K^+^ (87 mM) in the LBK medium to maintain cytoplasmic pH homeostasis. *E. coli* KNabc/pUC-SL38, KNabc/pUC18 as a negative control, KNabc/pUC-nhaD as a positive control of the sole Na^+^(Li^+^)/H^+^ antiporter (Wang et al., 2017), and KNabc/pUC-umpAB as a positive control of a Na^+^(Li^+^, K^+^)/H^+^ antiporter (Meng et al., 2017) were grown in the LBK media for the establishment of this hypothesis, or KCl-removed LBO media at the pH values from 7.0 to 9.0. As shown in **Figures 4A,B**, there is no significant difference between all *E. coli* KNabc transformants in the LBK media or LBO media at pH 7.0 and 7.5. In contrast, *E. coli* KNabc/pUC-SL38 and KNabc/pUC-umpAB could, but KNabc/pUC18 or KNabc/pUC-nhaD could not grow in the LBO medium at pH 8.0 (**Figure 4A**). More importantly, *E. coli* KNabc/pUC-SL38, similarly to KNabc/pUC-umpAB, showed the significantly higher growth in the LBK medium at pH 8.0 than KNabc/pUC18, similarly to KNabc/pUC-nhaD (**Figure 4B**). Even *E. coli* KNabc/pUC-SL38 could grow in the LBK medium at pH 8.5 while none of other *E. coli* KNabc transformants could grow under the same condition (**Figure 4B**). It should be pointed out that *E. coli* KNabc/pUC18 and KNabc/pUC-nhaD could grow at pH 8.0 in LBK medium containing 87 mM KCl (**Figure 4B**), but not at the same pH in the LBO medium without the addition of KCl (**Figure 4A**). That may be because that other K^+^/H^+^ antiporters such as MdtM (Holdsworth and Law, 2013) with the limited ability of maintaining alkaline pH homeostasis can’t support the growth of the host *E. coli* KNabc in the LBO medium only containing traces of contaminated K^+^ from 1.0% tryptone or 0.5% yeast extract when pH was increased to 8.0. In contrast, RDD and UmpAB with their stronger abilities of maintaining alkaline pH homeostasis could offer *E. coli* KNabc the growth under the same alkaline pH condition (**Figure 4A**). To more directly demonstrate that RDD may also function as a K^+^/H^+^ antiporter, the growth test was also carried out for KCl tolerance of the above-mentioned *E. coli* KNabc transformants. There is no significant difference between all *E. coli* KNabc transformants when KCl concentrations were varied from 0 to 0.8 M. However, *E. coli* KNabc/pUC-SL38, similarly to KNabc/pUC-umpAB, could grow significantly better in the presence of 0.9 M KCl than KNabc/pUC18, similarly to KNabc/pUC-nhaD (**Figure 4C**). More importantly, the sole *E. coli* KNabc/pUC-SL38, but not any other *E. coli* KNabc transformant, could grow in the presence of 1.0 M KCl (**Figure 4C**).

### Localization of RDD in the Cytoplasmic Membrane by Western Blot

**FIGURE 5:**
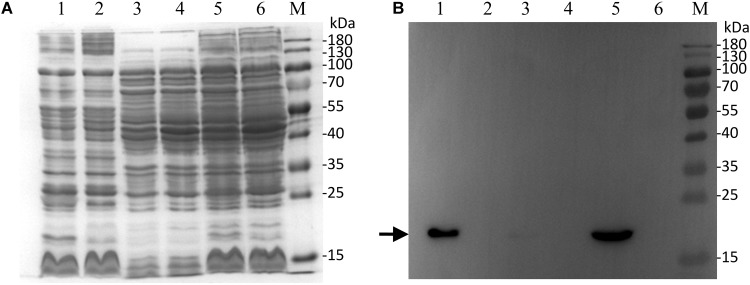
Western blot detection and localization of RDD in *Escherichia coli*. Membrane fraction, cytoplasmic one and cell extract from *E. coli* KNabc/pET22b-PRO-RDD (Lanes 1, 3, 5) and KNabc/pET22b (Lanes 2, 4, 6) were sampled, respectively, and then subjected to SDS-PAGE **(A)** and western blot **(B)** analyses. The position of target protein RDD fused with an N-terminal His_6_ tag is shown with a solid arrow.

To identify whether RDD is indeed a transmembrane protein, the detection and localization of RDD was carried out by western blot. The above-mentioned growth tests and sequencing analysis reveal that RDD was exactly fused with an N-terminal His_6_ tag and its native promoter took the place of the T7 promoter in pET22b. Therefore, the resultant construct pET22b-PRO-RDD, as well as pET22b, was selected for the preparation of the samples for the detection and localization of RDD, followed by the SDS-PAGE (**Figure 5A**) and western blot (**Figure 5B**) experiments. As shown in **Figure 5B**, very strong positive signals for RDD were detected in membrane fraction and cell extract from the cells of *E. coli* KNabc/pET22b-PRO-RDD, but not in those from KNabc/pET22b. Although there was also a weak signal in cytoplasmic fraction from KNabc/pET22b-PRO-RDD (**Figure 5B**), it may be caused by a trace amount of membrane fraction remaining in the cytoplasmic one due to non-complete separation of the former from the latter by ultracentrifugation.

### Determination of RDD as a Na^+^(Li^+^, K^+^)/H^+^ Antiporter

**FIGURE 6:**
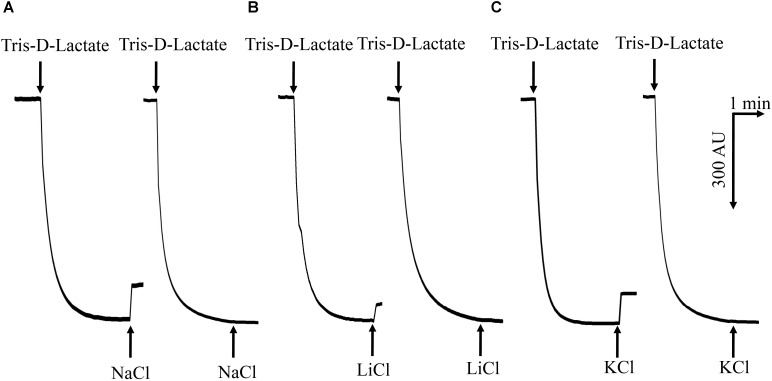
Assay for Na^+^(Li^+^, K^+^)/H^+^ antiport activity in the everted membrane vesicles. Na^+^/H^+^ antiport **(A)**, Li^+^/H^+^ antiport **(B)** and K^+^/H^+^ antiport **(C)** activities were measured in everted membrane vesicles prepared from cells of *E. coli* KNabc/pET22b-PRO-RDD (to the left) or KNabc/pET22b (to the right) by the French pressure cell method. The highest activity at pH 9.0 were shown as the representatives of each of them. At the time points indicated by downward arrows, Tris–D-lactic acid (final concentration at 10 mM) was added to the assay mixture to initiate fluorescence quenching. At the time points indicated by upward arrows, NaCl (final concentration at 10 mM), KCl (final concentration at 10 mM), or LiCl (final concentration at 10 mM) was added to the assay mixture. Fluorescence quenching is shown in arbitrary units.

**FIGURE 7:**
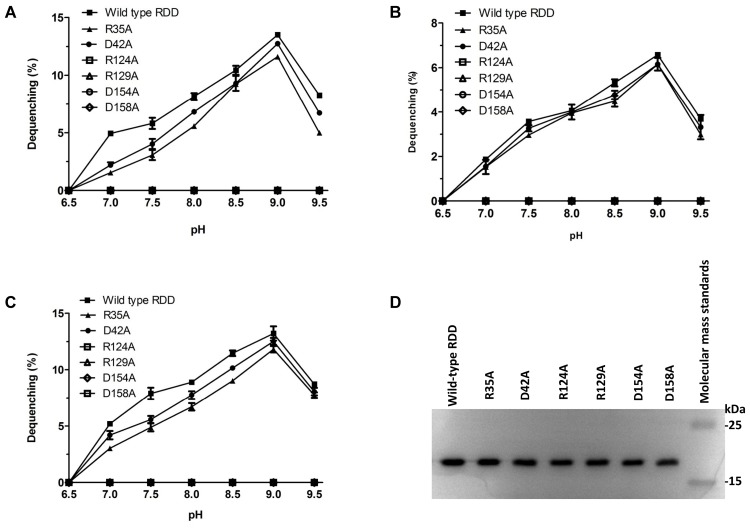
pH-dependent activity profile and western blot for RDD and its variants. The antiport activity was measured by the fluorescence dequenching method. Na^+^/H^+^ antiport activity **(A)**, Li^+^/H^+^ antiport activity **(B)** and K^+^/H^+^ antiport activity **(C)** for RDD and its variants were measured at the indicated pH values. Each value point represents the average of three independent determinations. Western blot detection **(D)** of wild-type RDD and its variants in the membrane fractions prepared from *E. coli* KNabc transformants.

To test whether RDD functions exactly as a Na^+^(Li^+^, K^+^)/H^+^ antiporter, *E. coli* KNabc cells carrying pET22b-PRO-RDD or pET22b were used for the preparation of everted membrane vesicles and then Na^+^(Li^+^, K^+^)/H^+^ antiport activity was measured by using the acridine orange fluorescence dequenching method. *E. coli* KNabc/pET22b showed no detectable Na^+^/H^+^, Li^+^/H^+^ or K^+^/H^+^ antiport activity (**Figure 6**) whereas KNabc/pET22b-PRO-RDD exhibited pH-dependent Na^+^/H^+^, Li^+^/H^+^ or K^+^/H^+^ antiport activity (**Figure 6**) at a wide range of pH between 6.5 and 9.5, with the highest activity at pH 9.0 (**Figure 7**).

### Evaluation of the Apparent Affinity of RDD for the Transported Cations

**FIGURE 8:**
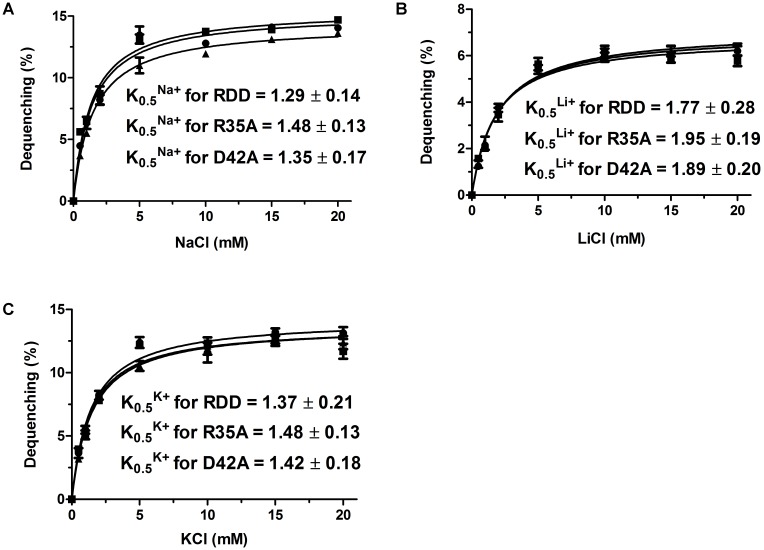
Calculation of K_0.5_ values of RDD and its variants for Na^+^, Li^+^ and K^+^. For the analysis of the apparent affinity of RDD or its variants for the transported cations, Na^+^/H^+^ **(A)**, Li^+^/H^+^ **(B)** and K^+^/H^+^ **(C)** antiport activities of *E. coli* KNabc transformant cells expressing RDD, R35A, and D42A were plotted as the respective functions of their corresponding cation concentrations. K_0.5_ values of RDD and its variants for Na^+^, Li^+^, and K^+^ were obtained, respectively, through the non-linear regression analysis with Prism 5.0. Each value point represents the average of three independent determinations.

To assess the apparent affinity of RDD for the transported cations, Na^+^/H^+^, Li^+^/H^+^ or K^+^/H^+^ antiport activity were measured at pH 9.0, respectively, by using everted membrane vesicles from KNabc/pET22b-PRO-RDD when the final concentrations of the corresponding cations were varied from 0 to 20 mM, followed by the calculation of K_0.5_ values of RDD as the representatives of half-maximal cation concentrations for Na^+^(Li^+^, K^+^)/H^+^ antiport activity. The respective K_0.5_ values of RDD are 1.29 ± 0.14 mM for Na^+^ (**Figure 8A**), 1.77 ± 0.28 mM for Li^+^ (**Figure 8B**) and 1.37 ± 0.21 mM for K^+^ (**Figure 8C**), indicating that RDD exhibits the apparent affinity with the order of Na^+^ > K^+^ > Li^+^_._

### Phylogenetic Analysis Between RDD and Its Homologs, Together With Known Na^+^/H^+^ Antiporters and Proteins With Na^+^/H^+^ Antiport Activity

**FIGURE 9:**
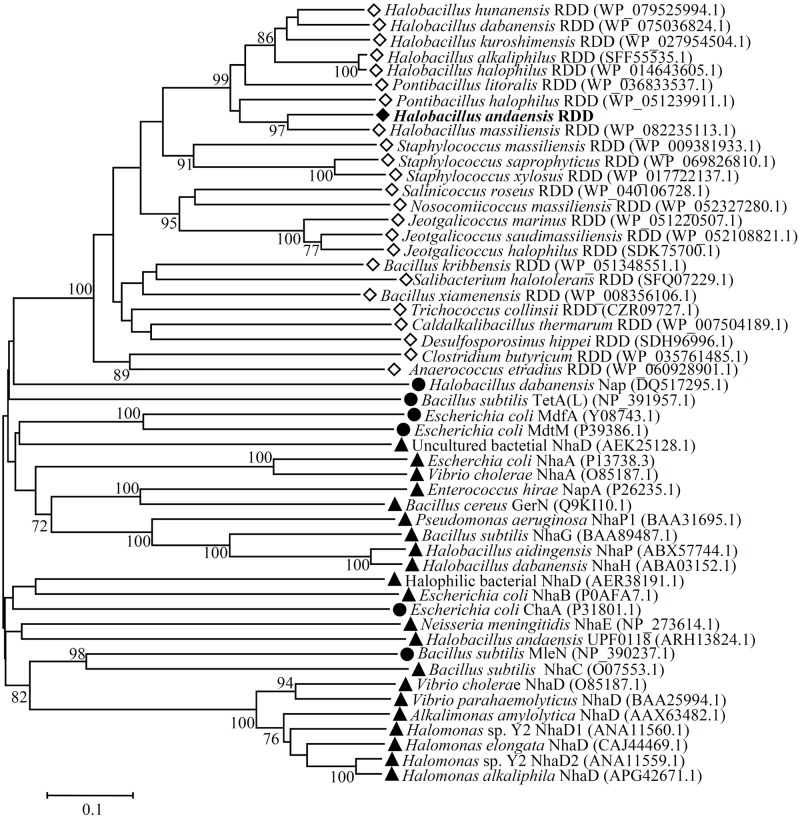
Neighbor-joining phylogenetic tree of RDD and its selected homologs, together with known Na^+^/H^+^ antiporters and proteins with Na^+^/H^+^ antiport activity. For the construction of phylogenetic tree, eight closest homologs with 51–74% identities, eight closer homologs with 41–50% identities, eight distant homologs with 26–39% identities, together with all representatives of known specific single-gene Na^+^/H^+^ antiporters and single-gene proteins with Na^+^/H^+^ antiport activity were selected. Accession.version numbers of selected homologs were shown in the parenthesis. Open diamond stands for putative RDD family proteins; filled diamond stands for RDD identified in this study; filled triangle stands for known specific single-gene Na^+^/H^+^ antiporters; filled circle stands for other single-gene proteins with the Na^+^/H^+^ antiport activity. Bootstrap values ≥70% (based on 1000 replications) are shown at branch points. Bar, 0.1 substitutions per amino acid residue position.

Since this RDD family protein exhibits Na^+^(Li^+^, K^+^)/H^+^ antiport activity, it is worthy of being further analyzed that RDD may represent a novel class of Na^+^(Li^+^, K^+^)/H^+^ antiporters. Therefore, a neighbor-joining phylogenetic tree was constructed to show the phylogenetic relationship of RDD with RDD family proteins and also identified single-gene proteins with Na^+^/H^+^ antiport activity. For this purpose, eight closest homologs with 51–74% identities, eight closer homologs with 41–50% identities, and eight distant homologs with 26–39% identities were selected. Also, all representatives of known specific single-gene Na^+^/H^+^ antiporters and other single-gene proteins with Na^+^/H^+^ antiport activity are inlcuded. RDD should belong to the RDD family since it is clustered with all its homologs with the bootstrap value of 100% (**Figure 9**). More importantly, RDD showed a quite distant relationship with not only known specific single-gene Na^+^(Li^+^)/H^+^ antiporters but also single-gene proteins with the Na^+^/H^+^ antiport activity.

### Recognition and Site-Directed Mutagenesis of the Conserved Arginine and Aspartate Residues

RDD family proteins are one category of uncharacterized transmembrane proteins containing three highly conserved amino acid residues, one arginine, and two aspartates, of which the arginine occurs at the N terminus of the first TMS in RDD family members and the first Asp occurs in the middle of this TMS. To identify the existence and exact positions of the above-mentioned three conserved residues in the amino acid sequence of RDD, RDD was aligned (**Figure 2A**) with all its 24 homologs clustered with the bootstrap value of 100% within the neighbor-joining phylogenetic tree. On the basis of multiple sequence alignment of RDD with the above-mentioned 24 homologs, a web-based amino acid sequence logo (**Figure 2B**) was also created in order to more clearly show the conservation of all the residues at the positions corresponding to those of RDD within the RDD family proteins. The 17 highly conserved residues including three arginines (R35, R124, and R129) and three aspartates (D42, D154, and D158) were found among the aligned homologs with a wide range of 26–74% identities (**Figures 2A,B**). As shown in **Figures 2A,B**, R35 should correspond with the arginine at the N terminus of the first TMS in RDD family proteins whereas D42 should correspond with the first Asp in the middle of this TMS. Combined with the sequence logo of 5, 254 RDD family members collected in the Pfam database shown at the website http://pfam.xfam.org/family/PF06271.10#tabview=tab4. The other highly conserved Asp was recognized to be D154, due to its being adjacent with one relatively conserved His (H) at the C terminus of RDD homologs (**Figures 2A,B**). Moreover, another two arginines and one aspartate were found to be fully conserved only between RDD and its selected homologs (**Figures 2A,B**), but not in the whole RDD family shown at the website http://pfam.xfam.org/family/PF06271.10#tabview=tab4.

Since R35, D42, and D154 are highly conserved among RDD family members, they are likely to be involved in the protein function of RDD. Also, R124, R129, and D158 are fully conserved between RDD and its selected homologs. Combined with the functional importance of charged aspartate and arginine residues for some identified Na^+^/H^+^ antiporters (Inoue et al., 1995; Noumi et al., 1997; Hellmer et al., 2003; Habibian et al., 2005; Hunte et al., 2005; Jiang et al., 2013b; Li et al., 2014), the above-mentioned six conserved residues were hypothesized to be functionally important residues of RDD and replaced by an alanine residue via a site-directed mutagenesis method for further probing their relevance with Na^+^(Li^+^, K^+^)/H^+^ antiport activity of RDD. The resultant variants were verified for the accuracy by re-sequencing and then designated R35A, D42A, R124A, R129A, D154A, and D158A (Supplementary Table S1), respectively. Furthermore, R124, R129, D154, and D158 were also replaced by the similar charged alkaline residue, lysine (K), or charged acidic residue, glutamic acid (E) (Supplementary Table S1), respectively, in order to further confirm the roles of the side chains of functionally critical residues in the activity of RDD. The resultant variants were also verified for the accuracy by re-sequencing and then designated R124K, R129K, D154E, and D158E (Table S1), respectively.

### Effect of RDD Variants by Site-Directed Mutagenesis on Bacterial Growth

**FIGURE 10:**
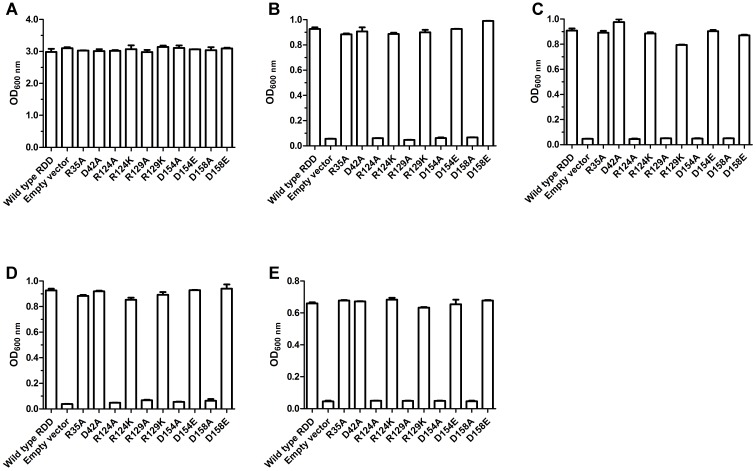
Effect of RDD variants by site-directed mutagenesis on bacterial growth. To identify the roles of the target residues on the protein function of RDD, *E. coli* KNabc expressing wild-type RDD or each of its all variants were grown in the LBK media at pH 7.0 containing no addition of NaCl or LiCl **(A)**, 0.2 M NaCl **(B)** or 5 mM LiCl **(C)**, or at pH 8.0 with the addition of 50 mM NaCl **(D)** or at pH 8.5 without the addition of NaCl **(E)**. The above mentioned cell growth was ended after 24 h and the values for OD_600 nm_ then evaluated. Each data point represents the average of three independent determinations.

To identify the roles of the above-mentioned residues on the protein function of RDD, the effects of substitutions in each of them by an alanine residue on the growth of *E. coli* KNabc were analyzed as the priority. *E. coli* KNabc expressing wild-type RDD or each of its all variants substituted by an alanine residue could show the normal growth in the LBK medium at pH 7.0 (**Figure 10A**). In contrast, the variants R124A, R129A, D154A, and D158A failed to complement the Na^+^-or-Li^+^-sensitive growth phenotype of *E. coli* KNabc in the LBK media containing 0.2 M NaCl (**Figure 10B**) or 5 mM LiCl (**Figure 10C**) at pH 7.0, or alkaline-pH-sensitive growth phenotype of *E. coli* KNabc in the LBK medium plus 50 mM NaCl at pH 8.0 (**Figure 10D**) or in the sole LBK medium at pH 8.5 (**Figure 10E**), indicating that these four residues play vital roles in Na^+^(Li^+^, K^+^)/H^+^ antiport activity of RDD. However, there was no growth difference between *E. coli* KNabc expressing wild-type RDD and R35A or D42A under any of the saline or alkaline stress conditions (**Figure 10**). Furthermore, the variants R124K, R129K, D154E, and D158E succeeded in recovering the Na^+^-or-Li^+^-sensitive (**Figures 10B,C**) or alkaline-pH-sensitive (**Figures 10D,E**) growth phenotypes of *E. coli* KNabc under the same stress conditions, confirming that the positive charges at the positions of No. 124 residue and No. 129 residue and the negative charges at the positions of No. 154 residue and No. 158 residue are responsible for Na^+^(Li^+^, K^+^)/H^+^ antiport activity of RDD.

### Measurements of RDD Variants Substituted by an Alanine Residue in the Cation Transport Activity

To confirm whether mutation in each of the above-mentioned six residues affects the Na^+^(Li^+^, K^+^)/H^+^ antiport activity of RDD, everted membrane vesicles were prepared from cells of *E. coli* KNabc expressing wild-type RDD as a positive control or each of its variants substituted by an alanine residue and then Na^+^(Li^+^, K^+^)/H^+^ antiport activity was measured at a wide range of pH between 6.5 and 9.5 by monitoring the dequenching of acridine orange fluorescence upon addition of NaCl, LiCl, or KCl. In comparison with wild-type RDD, the substitutions of R35 or D42 by an alanine residue led to almost no change of Na^+^(Li^+^, K^+^)/H^+^ antiport activity whereas the substitutions of R124, R129, D154, and D158 by an alanine residue absolutely abolished the Na^+^(Li^+^, K^+^)/H^+^ antiport activity (**Figures 7A–C**). Also, there is almost no difference of K_0.5_ values for Na^+^, K^+^ or Li^+^ between wild-type RDD and either of R35A or D42A (**Figures 8A–C**).

### Expression of RDD Variants Substituted by an Alanine Residue by the Western Blot

To rule out the possibility that the failed expressions of R124A, R129A, D154A, and D158A resulted in the loss of their antiport activities, the expressions of those four variants, as well as R35A, D42A and wild-type RDD as the positive controls, in the *E. coli* KNabc cells were analyzed by the western blot. As shown in **Figure 7D**, the expressions of RDD and each of its variants substituted by an alanine residue were detected with almost the same positive signals in membrane fractions prepared from *E. coli* KNabc transformants at the same amounts of total protein concentrations.

## Discussion

The RDD family has been functionally uncharacterized before this study. Here, we present the characterization of a member of this family with Na^+^(Li^+^, K^+^)/H^+^ antiport activity from the moderate halophile *H. andaensis* NEAU-ST10-40^T^. On the basis of the analyses of protein identity and phylogenetic relationship, we propose that RDD should represent a novel class of Na^+^(Li^+^, K^+^)/H^+^ antiporters, which is significantly distinct from all known single-gene Na^+^/H^+^ antiporters (Karpel et al., 1988; Pinner et al., 1992; Waser et al., 1992; Nakamura et al., 1996; Ito et al., 1997; Nozaki et al., 1998; Utsugi et al., 1998; Gouda et al., 2001; Southworth et al., 2001; Herz et al., 2003; Liu et al., 2005; Kurz et al., 2006; Yang et al., 2006c; Sousa et al., 2013; Zhang et al., 2014; Cui et al., 2016; Wang et al., 2017), double-gene (Jiang et al., 2013a; Meng et al., 2017), or multiple-gene Na^+^/H^+^ antiporters (Hamamoto et al., 1994; Hiramatsu et al., 1998; Putnoky et al., 1998; Kosono et al., 1999; Jiang et al., 2004; Yang et al., 2006a; Dzioba-Winogrodzki et al., 2009; Yamaguchi et al., 2009; Meng et al., 2014), or other proteins with Na^+^/H^+^ antiport activity (Cheng et al., 1994; Wei et al., 2000; Lewinson et al., 2004; Yang et al., 2006b; Holdsworth and Law, 2013). Combining the conservation of three arginines and three aspartates among the whole family or between RDD and its selected homologs with the functional importance of charged arginine and aspartate residues for some identified Na^+^/H^+^ antiporters (Inoue et al., 1995; Noumi et al., 1997; Hellmer et al., 2003; Habibian et al., 2005; Hunte et al., 2005; Jiang et al., 2013b; Li et al., 2014), we analyzed the roles of the above-mentioned six residues in the cation-transporting activity through site-directed mutagenesis and found that D154, of those three highly conserved residues among the whole RDD family, and all three fully conserved residues including R124, R129, and D158 between RDD and its selected homologs, are indispensable for Na^+^(Li^+^, K^+^)/H^+^ antiport activity, whereas neither R35 nor D42 of those three highly conserved residues among the whole RDD family is involved in Na^+^(Li^+^, K^+^)/H^+^ antiport activity. To the best of our knowledge, this is the first report on the characterization of a representative of RDD family proteins and functional analysis of its conserved arginine/aspartate residues. As a dual representative of a novel class of Na^+^(Li^+^, K^+^)/H^+^ antiporters and RDD family proteins, the characterization of RDD and analysis of its functionally important residues will positively contribute to the knowledge of the cation-transporting mechanisms of this novel antiporter and the roles of highly conserved arginine/aspartate residues in the functions of RDD family proteins.

RDD was predicted to be a transmembrane protein with three putative TMSs (**Figure 2A**), which was established by the localization of RDD by western blot in the cytoplasmic membranes of *E. coli* KNabc (**Figure 5**). Growth tests (**Figures 3**, **4**) and Na^+^(Li^+^, K^+^)/H^+^ antiport assay (**Figures 6**–**8**) reveal that RDD is likely to function as both an Na^+^ (Li^+^)/H^+^ antiporter and a K^+^/H^+^ antiporter. A careful protein alignment using BLASTP (Altschul et al., 1990) at the NCBI website reveals that RDD has no identity with all known single-gene, double-gene, or multiple-gene Na^+^/H^+^ antiporters or proteins with Na^+^/H^+^ antiport activity. Phylogenetic analysis confirms that there is a quite distant relationship between RDD and all known single-gene Na^+^/H^+^ antiporters and proteins with Na^+^/H^+^ antiport activity (**Figure 9**). Therefore, we propose that RDD should represent a novel class of Na^+^(Li^+^, K^+^)/H^+^ antiporters.

As shown at the website http://pfam.xfam.org/family/PF06271.10#tabview=tab0, 5,254 functionally uncharacterized transmembrane proteins contain three highly conserved amino acid residues, one arginine and two aspartates, which are therefore classified into one category designated RDD family in the Pfam database. The sole additional known information is that this family of proteins contain three putative TMSs and the arginine of “arginine–aspartate–aspartate” occurs at the N terminus of the first TMS and the first aspartate occurs in the middle of this TMS. Protein alignment showed the existence of the above-mentioned three highly conserved residues (R35, D42, and D154) in the amino acid sequence of RDD (**Figure 2A**), which was more clearly presented in a web-based amino acid sequence logo on the basis of multiple sequence alignment of RDD with its selected 24 homologs (**Figure 2B**). Therefore, RDD from *H. andaensis* should belong to RDD family. That was confirmed by the phylogenetic analysis result that RDD and its selected homologs clustered with the bootstrap value of 100%. It should be emphasized that we downloaded RDD homologs from as many different genera or species at the NCBI website as possible in order to guarantee the representatives of RDD homologs within the respective identity ranges of 51–74%, 41–50%, and 26–39%. Also, we warranted that the difference of identity is above at least 0.5% (mostly 2–3%) to cover all the representatives of RDD homologs within the above-mentioned three identity ranges. Therefore, the function of RDD is likely to reflect those of this family of proteins at least within a wide range of 26–74% identities. In this study, we at least present the first report on the function of RDD as a Na^+^(Li^+^, K^+^)/H^+^ antiporter. This novel finding triggers the knowledge of the function of RDD family proteins that have not been functionally characterized as yet.

More attention should be paid to the relationship between the above-mentioned three highly conserved residues (R35, D42, and D154 in RDD) and the function of RDD family proteins, since they are highly conserved among the whole RDD family. The conserved charged aspartate and arginine residues have been widely reported to play vital roles in the functions of some identified Na^+^/H^+^ antiporters (Inoue et al., 1995; Noumi et al., 1997; Hellmer et al., 2003; Habibian et al., 2005; Hunte et al., 2005; Jiang et al., 2013b; Li et al., 2014). Considering that another two arginine residues (R124 and R129) and one aspartate residue (D158) are fully conserved between RDD and its selected homologs within a wide range of 26–74% identities, it is also worthy of being explored whether they are related with Na^+^(Li^+^, K^+^)/H^+^ antiport activity of RDD. Therefore, we used site-directed mutagenesis to replace the above-mentioned six residues of RDD with an alanine residue to analyze their functional importance for RDD. Of the three conserved residues among the whole RDD family, the sole D154A failed to complement with the host *E. coli* KNabc under the tested saline or alkaline stress conditions (**Figure 10**) and absolutely abolished the Na^+^(Li^+^, K^+^)/H^+^ antiport activity (**Figures 7A–C**), indicating that D154 is indispensable for Na^+^(Li^+^, K^+^)/H^+^ antiport activity of RDD. Unexpectedly, neither R35A nor D42A showed the influence on the normal growth of the host *E. coli* KNabc under the tested saline or alkaline stress conditions (**Figure 10**) and retained the similar Na^+^(Li^+^, K^+^)/H^+^ antiport activity (**Figures 7A–C**) and apparent affinity for the transported cations to those for wild-type RDD (**Figure 8**), revealing that neither R35 nor D42 is involved in the Na^+^(Li^+^, K^+^)/H^+^ antiport activity of RDD. However, each of R124A, R129A, or D158A failed to complement with the host *E. coli* KNabc under the tested saline or alkaline stress conditions (**Figure 10**) and absolutely abolished the Na^+^(Li^+^, K^+^)/H^+^ antiport activity (**Figures 7A–C**), indicating that R124, R129, and D158 are indispensable for Na^+^(Li^+^, K^+^)/H^+^ antiport activity of RDD. It should be stressed that immunochemical localization reveal that the missing activity of the R124A, R129A, D154A, and D158A variants can’t be attributed to the inabilities of their expressions (**Figure 7D**). Furthermore, R124K, R129K, D154E, and D158E recovered their abilities to complement with the host *E. coli* KNabc under the tested saline or alkaline stress conditions (**Figure 10**), reflecting the roles of the side chains of functionally critical residues in the activity of RDD. Taken together, we speculate that the carboxyl group in the side chains of D154 and D158 may be responsible for the cation binding or translocation, which are similar to the roles of the negatively charged aspartate residues as the active sites of identified Na^+^/H^+^ antiporters such as Ec*_*NhaA (Inoue et al., 1995; Habibian et al., 2005; Hunte et al., 2005), Hd_NhaH (Jiang et al., 2013b), and Mj_NhaP1 (Hellmer et al., 2003) for the transported cations. Despite the inability of R124 or R129 directly binding with the cations, the positive charges in their side chains may be used for corresponding to the charge-induced subtle and fast conformational changes, which are similar to the roles of the positively charged arginine or lysine residues such as R425 (Li et al., 2014) of mammalian NHE1 or K300 (Hunte et al., 2005) of Ec*_*NhaA in the compensation for the TMS dipoles. Although R35 and D42 are highly conserved among the whole RDD family, they were established not to be involved in the function of RDD as a Na^+^(Li^+^, K^+^)/H^+^ antiporter. However, it’s still very interesting why they are commonly shared by more than 5, 000 RDD family members. Also, it needs to be better explained possibly on the basis of the structural analysis whether the positive charges in the side chains of R124 and R129 act as the key compensating residues for the TMS dipoles and whether the carboxyl groups in the side chains of D154 and D158 are directly involved in the cation transportation of RDD. To illuminate the above-mentioned issues of interest, we attempted to construct the modeled structure of RDD based on its amino acid sequence using the Phyre 2 molecular modeling server at the website http://www.sbg.bio.ic.ac.uk/~phyre2/html/page.cgi?id=index. However, no modeled structure with the high confidence scores was available. The insight to these interesting points will seem to depend on the crystal structure analysis of RDD in future studies. Moreover, since RDD functions as a novel Na^+^(Li^+^, K^+^)/H^+^ antiporter, a more detailed exploration for the structure–function relationship of RDD will positively contribute to the knowledge of the novel cation-transporting mechanisms by Na^+^/H^+^ antiporters. Therefore, whether other fully conserved charged or polar residues such as Y87, T92, K101, E125, Y140, and T159 and relatively highly conserved charged or polar residues such as T98 and H153 were involved in Na^+^(Li^+^, K^+^)/H^+^ antiport activity of RDD should also be further analyzed via a more detailed site-directed mutagenesis in future studies.

## Author Contributions

JJ and LS designed the experiments, prepared the figures and tables, performed the data analysis, and revised the manuscript. The experiments were carried out by LS, HA-M, JC, HC, TX, LM, ZZ, and FM. The results of the experiments were interpreted by LS, HA-M and FM, and discussed by all authors. The manuscript was drafted by LS and HA-M. The final version was approved by all authors.

## Conflict of Interest Statement

The authors declare that the research was conducted in the absence of any commercial or financial relationships that could be construed as a potential conflict of interest.

## References

[B1] Altschul S. F. Gish W. Miller W. Myers E. W. Lipman D. J. (1990). Basic local alignment search tool. *J. Mol. Biol.* 215 403–410. 10.1016/S0022-2836(05)80360-2 2231712

[B2] Cheng B. Meng Y. Cui Y. Li C. Tao F. Yin H. (2016). Alkaline response of a halotolerant alkaliphilic *Halomonas* strain and functional diversity of its Na+(K+)/H+ antiporters. *J. Biol. Chem.* 291 26056–26065. 10.1074/jbc.M116.751016 27777302 PMC5207076

[B3] Cheng J. Guffanti A. A. Krulwich T. A. (1994). The chromosomal tetracycline resistance locus of *Bacillus subtilis* encodes a Na+/H+ antiporter that is physiologically important at elevated pH. *J. Biol. Chem.* 269 27365–27371. 7961647

[B4] Crooks G. E. Hon G. Chandonia J. M. Brenner S. E. (2004). WebLogo: a sequence logo generator. *Genome Res.* 14 1188–1190. 10.1101/gr.849004 15173120 PMC419797

[B5] Cui Y. Cheng B. Meng Y. Li C. Yin H. Xu P. (2016). Expression and functional analysis of two NhaD type antiporters from the halotolerant and alkaliphilic Halomonas sp. Y2. *Extremophiles* 20 1–9. 10.1007/s00792-016-0852-8 27315164

[B6] Dong P. Wang L. Song N. Yang L. Chen J. Yan M. (2017). A UPF0118 family protein with uncharacterized function from the moderate halophile *Halobacillus andaensis* represents a novel class of Na+(Li+)/H+ antiporter. *Sci. Rep.* 7:45936. 10.1038/srep45936 28374790 PMC5379678

[B7] Dzioba-Winogrodzki J. Winogrodzki O. Krulwich T. A. Boin M. A. Dibrov P. (2009). The *Vibrio cholerae* Mrp system: cation/proton antiport properties and enhancement of bile salt resistance in a heterologous host. *J. Mol. Microbiol. Biotechnol.* 16 176–186. 10.1159/000119547 18311075 PMC2640445

[B8] Gouda T. Kuroda M. Hiramatsu T. Nozaki K. Kuroda T. Mizushima T. (2001). nhaG Na+/H+ antiporter gene of *Bacillus subtilis* ATCC9372, which is missing in the complete genome sequence of strain 168, and properties of the antiporter. *J. Biochem.* 130 711–717. 10.1093/oxfordjournals.jbchem.a003038 11686935

[B9] Green M. R. Sambrook J. (2012). *Molecular Cloning: A Laboratory Manual IV*. New York, NY: Cold Spring Harbor Laboratory Press.

[B10] Habibian R. Dzioba J. Barrett J. Galperin M. Y. Loewen P. C. Dibrov P. (2005). Functional analysis of conserved polar residues in Vc-NhaD, Na+/H+ antiporter of *Vibrio cholerae*. *J. Biol. Chem.* 280 39637–39643. 10.1074/jbc.M509328200 16186100

[B11] Hamamoto T. Hashimoto M. Hino M. Kitada M. Seto Y. Kudo T. (1994). Characterization of a gene responsible for the Na+/H+ antiporter system of alkalophilic *Bacillus* species strain C-125. *Mol. Microbiol.* 14 939–946. 10.1111/j.1365-2958.1994.tb01329.x 7715455

[B12] Hellmer J. Teubner A. Zeilinger C. (2003). Conserved arginine and aspartate residues are critical for function of MjNhaP1, a Na+/H+ antiporter of *M. jannaschii*. *FEBS Lett.* 542 32–36. 10.1016/S0014-5793(03)00332-6 12729893

[B13] Herz K. Vimont S. Padan E. Berche P. (2003). Roles of NhaA, NhaB, and NhaD Na+/H+ antiporters in survival of *Vibrio cholerae* in a saline environment. *J. Bacteriol.* 185 1236–1244. 10.1128/JB.185.4.1236-1244.2003 12562793 PMC142861

[B14] Hiramatsu T. Kodama K. Kuroda T. Mizushima T. Tsuchiya T. (1998). A putative multisubunit Na+/H+ antiporter from *Staphylococcus aureus*. *J. Bacteriol.* 180 6642–6648. 9852009 10.1128/jb.180.24.6642-6648.1998 PMC107768

[B15] Holdsworth R. S. Law J. C. (2013). Multidrug resistance protein MdtM adds to the repertoire of antiporters involved in alkaline pH homeostasis in *Escherichia coli*. *BMC Microbiol.* 13:113. 10.1186/1471-2180-13-113 23701827 PMC3668916

[B16] Hunte C. Screpanti E. Venturi M. Rimon A. Padan E. Michel H. (2005). Structure of a Na+/H+ antiporter and insights into mechanism of action and regulation by pH. *Nature* 435 1197–1202. 10.1038/nature03692 15988517

[B17] Inoue H. Noumi T. Tsuchiya T. Kanazawa H. (1995). Essential aspartic acid residues, Asp-133, Asp-163 and Asp-164, in the transmembrane helices of a Na+/H+ antiporter (NhaA) from *Escherichia coli*. *FEBS Lett.* 363 264–268. 10.1016/0014-5793(95)00331-3 7737413

[B18] Ito M. Guffanti A. A. Oudega B. Krulwich T. A. (1999). mrp, a multigene, multifunctional locus in *Bacillus subtilis* with roles in resistance to cholate and to Na+ and in pH homeostasis. *J. Bacteriol.* 181 2394–2402. 10198001 10.1128/jb.181.8.2394-2402.1999 PMC93663

[B19] Ito M. Guffanti A. A. Zemsky J. Ivey D. M. Krulwich T. A. (1997). Role of the nhaC-encoded Na+/H+ antiporter of alkaliphilic *Bacillus firmus* OF4. *J. Bacteriol.* 179 3851–3857. 10.1128/jb.179.12.3851-3857.1997 9190799 PMC179192

[B20] Ivey D. M. Guffanti A. A. Zemsky J. Pinner E. Karpel R. Padan E. (1993). Cloning and characterization of a putative Ca2+/H+ antiporter gene from *Escherichia coli* upon functional complementation of Na+/H+ antiporter-deficient strains by the overexpressed gene. *J. Biol. Chem.* 268 11296–11303. 8496184

[B21] Jiang J. Wang L. Zhang H. Wu H. Huang H. Yang L. (2013a). Putative paired small multidrug resistance family proteins PsmrAB, the homolog of YvdSR, actually function as a novel two-component Na+/H+ antiporter. *FEMS Microbiol. Lett.* 338 31–38. 10.1111/1574-6968.12008 22978536

[B22] Jiang J. Wang L. Zou Y. Lu W. Zhao B. Zhang B. (2013b). Identification of important charged residues for alkali cation exchange or pH regulation of NhaH, a Na+/H+ antiporter of *Halobacillus dabanensis*. *Biochim. Biophys. Acta* 1828 997–1003. 10.1016/j.bbamem.2012.11.015 23196349

[B23] Jiang J. Wei W. Du B. Li X. Wang L. Yang S. (2004). Salt-tolerance genes involved in cation efflux and osmoregulation of *Sinorhizobium fredii* RT19 detected by isolation and characterization of Tn5 mutants. *FEMS Microbiol. Lett.* 239 139–146. 10.1016/j.femsle.2004.08.029 15451112

[B24] Karpel R. Olami Y. Taglicht D. Schuldiner S. Padan E. (1988). Sequencing of the gene ant which affects the Na+/H+ antiporter activity in *Escherichia coli*. *J. Biol. Chem.* 263 10408–10414. 2839489

[B25] Kosono S. Morotomi S. Kitada M. Kudo T. (1999). Analyses of a *Bacillus subtilis* homologue of the Na+/H+ antiporter gene which is important for pH homeostasis of alkaliphilic *Bacillus* sp. C-125. *Biochim. Biophys. Acta* 1409 171–175. 10.1016/S0005-2728(98)00157-1 9878723

[B26] Krulwich T. A. Hicks D. B. Ito M. (2009). Cation/proton antiporter complements of bacteria: why so large and diverse? *Mol. Microbiol.* 74 257–260. 10.1111/j.1365-2958.2009.06842.x 19682259 PMC2765581

[B27] Kurz M. Brünig A. N. Galinski E. A. (2006). NhaD type sodium/proton-antiporter of *Halomonas elongata*: a salt stress response mechanism in marine habitats? *Saline Syst.* 2:10. 16872527 10.1186/1746-1448-2-10 PMC1552076

[B28] Lewinson O. Padan E. Bibi E. Kaback H. R. (2004). Alkalitolerance: a biological function for a multidrug transporter in pH homeostasis. *Proc. Natl. Acad. Sci. U.S.A.* 101 14073–14078. 10.1073/pnas.0405375101 15371593 PMC521123

[B29] Li X. Ma Y. Fliegel L. (2014). Functional role of arginine 425 in the mammalian Na+/H+ exchanger. *Biochem. Cell Biol.* 92 541–546. 10.1139/bcb-2014-0070 25350536

[B30] Liu J. Xue Y. Wang Q. Wei Y. Swartz T. H. Hicks D. B. (2005). The activity profile of the NhaD-Type Na+(Li+)/H+ antiporter from the soda lake haloalkaliphile *Alkalimonas amylolytica* is adaptive for the extreme environment. *J. Bacteriol.* 187 7589–7595. 10.1128/JB.187.22.7589-7595.2005 16267283 PMC1280297

[B31] Lowry O. H. Rosebrough N. J. Farr A. L. Randall R. J. (1951). Protein measurement with the Folin phenol reagent. *J. Biol. Chem.* 193 265–275. 14907713

[B32] Meng L. Hong S. Liu H. Huang H. Sun H. Xu T. (2014). Cloning and identification of Group 1 mrp operon encoding a novel monovalent cation/proton antiporter system from the moderate halophile *Halomonas zhaodongensis*. *Extremophiles* 18 963–972. 10.1007/s00792-014-0666-5 24996797

[B33] Meng L. Meng F. Zhang R. Zhang Z. Dong P. Sun K. (2017). Characterization of a novel two-component Na+(Li+, K+)/H+ antiporter from *Halomonas zhaodongensis*. *Sci. Rep.* 7:4221. 10.1038/s41598-017-04236-0 28652569 PMC5484666

[B34] Mesbah N. M. Cook G. M. Wiegel J. (2009). The halophilic alkalithermophile *Natranaerobius thermophilus* adapts to multiple environmental extremes using a large repertoire of Na+(K+)/H+ antiporters. *Mol. Microbiol.* 74 270–281. 10.1111/j.1365-2958.2009.06845.x 19708921 PMC2764116

[B35] Nakamura T. Enomoto H. Unemoto T. (1996). Cloning and sequencing of nhaB gene encoding an Na+/H+ antiporter from *Vibrio alginolyticus*. *Biochim. Biophys. Acta* 1275 157–160. 10.1016/0005-2728(96)00034-5 8695633

[B36] Noumi T. Inoue H. Sakurai T. Tsuchiya T. Kanazawa H. (1997). Identification and characterization of functional residues in a Na+/H+ antiporter (NhaA) from *Escherichia coli* by random mutagenesis. *J. Biochem.* 121 661–670. 10.1093/oxfordjournals.jbchem.a021637 9163515

[B37] Nozaki K. Inaba K. Kuroda T. Tsuda M. Tsuchiya T. (1996). Cloning and sequencing of the gene for Na+/H+ antiporter of *Vibrio parahaemolyticus*. *Biochem. Biophys. Res. Commun.* 222 774–779. 10.1006/bbrc.1996.0820 8651921

[B38] Nozaki K. Kuroda T. Mizushima T. Tsuchiya T. (1998). A new Na+/H+ antiporter, NhaD, of *Vibrio parahaemolyticus*. *Biochim. Biophys. Acta* 1369 213–220. 10.1016/S0005-2736(97)00223-X 9518619

[B39] Oren A. (1999). Bioenergetic aspects of halophilism. *Microbiol. Mol. Biol. Rev.* 63 334–348. 10357854 10.1128/mmbr.63.2.334-348.1999 PMC98969

[B40] Padan E. Bibi E. Ito M. Krulwich T. A. (2005). Alkaline pH homeostasis in bacteria: new insights. *Biochim. Biophys. Acta* 1717 67–88. 10.1016/j.bbamem.2005.09.010 16277975 PMC3072713

[B41] Padan E. Schuldiner S. (1994). Molecular physiology of Na+/H+ antiporters, key transporters in circulation of Na+ and H+ in cells. *Biochim. Biophys. Acta* 1185 129–151. 10.1016/0005-2728(94)90204-6 8167133

[B42] Padan E. Venturi M. Gerchman Y. Dover N. (2001). Na+/H+ antiporters. *Biochim. Biophys. Acta* 1505 144–157. 10.1016/S0005-2728(00)00284-X 11248196

[B43] Pinner E. Padan E. Schuldiner S. (1992). Cloning, sequencing, and expression of the nhaB gene, encoding a Na+/H+ antiporter in *Escherichia coli*. *J. Biol. Chem.* 267 11064–11068. 1317851

[B44] Putnoky P. Kereszt A. Nakamura T. Endre G. Grosskopf E. Kiss P. (1998). The pha gene cluster of *Rhizobium meliloti* involved in pH adaptation and symbiosis encodes a novel type of K+ efflux system. *Mol. Microbiol.* 28 1091–1101. 10.1046/j.1365-2958.1998.00868.x 9680201

[B45] Quinn M. J. Resch C. T. Sun J. Lind E. J. Dibrov P. Häse C. C. (2012). NhaP1 is a K+(Na+)/H+ antiporter required for growth and internal pH homeostasis of *Vibrio cholerae* at low extracellular pH. *Microbiology* 158 1094–1105. 10.1099/mic.0.056119-0 22241048 PMC3949420

[B46] Radchenko M. V. Tanaka K. Waditee R. Oshimi S. Matsuzaki Y. Fukuhara M. (2006). Potassium/proton antiport system of *Escherichia coli*. *J. Biol. Chem.* 281 19822–19829. 10.1074/jbc.M600333200 16687400

[B47] Reese M. G. (2001). Application of a time-delay neural network to promoter annotation in the *Drosophila melanogaster* genome. *J. Comput. Chem.* 26 51–56. 10.1016/S0097-8485(01)00099-7 11765852

[B48] Rosen B. P. (1986). Ion extrusion system in *Escherichia coli*. *Methods Enzymol.* 125 328–336. 10.1016/S0076-6879(86)25028-4 2423844

[B49] Saier M. H. Jr. Eng B. H. Fard S. Garg J. Haggerty D. A. Hutchinson W. J. (1999). Phylogenetic characterization of novel transport protein families revealed by genome analyses. *Biochim. Biophys. Acta* 1422 1–56. 10.1016/S0304-4157(98)00023-9 10082980

[B50] Saier M. H. Jr. Reddy V. S. Tsu B. V. Ahmed M. S. Li C. Moreno-Hagelsieb G. (2016). The Transporter classification database (TCDB): recent advances. *Nucleic Acids Res.* 44 D372–D379. 10.1093/nar/gkv1103 26546518 PMC4702804

[B51] Saitou N. Nei M. (1987). The neighbor-joining method: a new method for reconstructing phylogenetic trees. *Mol. Biol. Evol.* 4 406–425. 3447015 10.1093/oxfordjournals.molbev.a040454

[B52] Slonczewski J. L. Fujisawa M. Dopson M. Krulwich T. A. (2009). Cytoplasmic pH measurement and homeostasis in bacteria and archaea. *Adv. Microb. Physiol.* 55 1–79. 10.1016/S0065-2911(09)05501-5 19573695

[B53] Sousa P. M. F. Videira M. A. M. Vorburger T. Silva S. T. N. Moir J. W. Steuber J. (2013). The novel NhaE-type Na+/H+ antiporter of the pathogenic bacterium *Neisseria meningitidis*. *Arch. Microbiol.* 195 211–217. 10.1007/s00203-012-0856-4 23208205

[B54] Southworth T. W. Guffanti A. A. Moir A. Krulwich T. A. (2001). GerN, an endospore germination protein of *Bacillus cereus*, is an Na+/H+-K+ antiporter. *J. Bacteriol.* 183 5896–5903. 10.1128/JB.183.20.5896-5903.2001 11566988 PMC99667

[B55] Thompson J. D. Gibson T. J. Plewniak F. Jeanmougin F. Higgins D. G. (1997). The ClustalX windows interface: flexible strategies for multiple sequence alignment aided by quality analysis tools. *Nucleic Acids Res.* 25 4876–4882. 10.1093/nar/25.24.4876 9396791 PMC147148

[B56] Utsugi J. Inaba K. Kuroda T. Tsuda M. Tsuchiya T. (1998). Cloning and sequencing of a novel Na+/H+ antiporter gene from *Pseudomonas aeruginosa*. *Biochim. Biophys. Acta* 1398 330–334. 10.1016/S0167-4781(98)00058-X 9655928

[B57] Ventosa A. Nieto J. J. Oren A. (1998). Biology of moderately halophilic aerobic bacteria. *Microbiol. Mol. Biol. Rev.* 62 504–544. 9618450 10.1128/mmbr.62.2.504-544.1998 PMC98923

[B58] Wang K. Zhang L. Yang Y. Pan Y. Meng L. Liu H. (2015). *Halobacillus andaensis* sp. nov., a moderately halophilic bacterium isolated from saline and alkaline soil. *Int. J. Syst. Evol. Microbiol.* 65 1908–1914. 10.1099/ijs.0.000198 25795064

[B59] Wang Y. Song N. Yang L. Abdel-Motaal H. Zhang R. Zhang Z. (2017). A novel NhaD-type Na+/H+ antiporter from the moderate halophile and alkaliphile *Halomonas alkaliphila*. *Can. J. Microbiol.* 63 596–607. 10.1139/cjm-2017-0104 28329448

[B60] Waser M. Hessbienz D. Davies K. Solioz M. (1992). Cloning and disruption of a putative NaH-antiporter gene of *Enterococcus hirae*. *J. Biol. Chem.* 267 5396–5400. 1312090

[B61] Wei Y. Guffanti A. A. Ito M. Krulwich T. A. (2000). *Bacillus subtilis* YqkI is a novel malic/Na+-lactate antiporter that enhances growth on malate at low protonmotive force. *J. Biol. Chem.* 275 30287–30292. 10.1074/jbc.M001112200 10903309

[B62] Yamaguchi T. Tsutsumi F. Putnoky P. Fukuhara M. Nakamura T. (2009). pH-dependent regulation of the multi-subunit cation/proton antiporter Pha1 system from *Sinorhizobium meliloti*. *Microbiology* 155 2750–2756. 10.1099/mic.0.028563-0 19460820

[B63] Yang L. Jiang J. Wei W. Zhang B. Wang L. Yang S. (2006a). The *pha2* gene cluster involved in Na+ resistance and adaption to alkaline pH in *Sinorhizobium fredii* RT19 encodes a monovalent cation/proton antiporter. *FEMS Microbiol. Lett.* 262 172–177. 16923072 10.1111/j.1574-6968.2006.00385.x

[B64] Yang L. Jiang J. Zhang B. Zhao B. Wang L. Yang S. (2006b). A primary sodium pump gene of the moderate halophile *Halobacillus dabanensis* exhibits secondary antiporter properties. *Biochem. Biophys. Res. Commun.* 346 612–617. 16774742 10.1016/j.bbrc.2006.05.181

[B65] Yang L. Jiang J. Zhao B. Zhang B. Feng D. Lu W. (2006c). A Na+/H+ antiporter gene of the moderately halophilic bacterium *Halobacillus dabanensis* D-8T: cloning and molecular characterization. *FEMS Microbiol. Lett.* 255 89–95. 16436066 10.1111/j.1574-6968.2005.00055.x

[B66] Zhang H. Wang Z. Wang L. Mu R. Zou Z. Yuan K. (2014). Cloning and identification of a novel NhaD-type Na+/H+ antiporter from metagenomic DNA of the halophilic bacteria in soil samples around Daban Salt Lake. *Extremophiles* 18 89–98. 10.1007/s00792-013-0600-2 24297704

