# Supplementary material for: Characterization of a Functionally Unknown Arginine–Aspartate–Aspartate Family Protein From Halobacillus andaensis and Functional Analysis of Its Conserved Arginine/Aspartate Residues
Source: Front Microbiol. 2018 Apr 25;9:807. doi: 10.3389/fmicb.2018.00807 (PMC5996927; doi:10.3389/fmicb.2018.00807)
Supplement: Supplementary file 1 [file Table_1.PDF]

Supplementary Material

Characterization of a functionally unknown arginine-aspartate-aspartate family protein from *Halobacillus andaensis* and functional analysis of its conserved arginine/aspartate residues

Li Shao, Heba Abdel-Motaal, Jin Chen, Huiwen Chen, Tong Xu, Lin Meng, Zhenglai Zhang, Fankui Meng, Juquan Jiang*

*** Correspondence:** Corresponding Author: jjqdainty@163.com

Supplementary Table 1. Oligonucleotide primers used for subcloning and site-directed mutagenesis of *rdd* gene, and cloning of its native promoter.

Primer ID	Primer sequence	Primer usage
SppA_F	5'-CATATGGATCAAATCTATGCATCTGA-3' (*Nde*I, underlined)	for subcloning of 5'-end truncated *sppA* gene fused in frame with an N-terminal His_6 tag in pET19b
SppA_R	5'-GGATCCTTACTCAGAATACATGTACA-3' (*Bam*HI, underlined)	
RDD_F	5'-CATATGGATACAGTAGAACCCAC-3' (*Nde*I, underlined)	for subcloning of wild-type *rdd* gene fused in frame with an N-terminal His_6 tag in pET28b-RDD
RDD_R	5'-CTCGAGTTAATCTACAGAAGATTCAA-3' (*Xho*I, underlined)	
Promoter_F	5'-AGATCTGATGAAGTGATTGAAATTG-3' (*Bgl*II, underlined)	for cloning of the native promoter of wild-type *rdd* gene in pET22b-PRO-RDD
Promoter_R	5'-CCATGGCTAATCCCTCCTTTACTCA-3' (*Nco*I, underlined)	
R35A_F	5'-GCTGGTTTTGTTCCAGCCTTTTTTGCT-3' (Mutagenic bases, underlined)	for the codon change of No. 35 residue from CGC for Arg to GCC for Ala
R35A_R	5'-GCTGGAACAAAACCAGCATATACAATG-3' (Mutagenic bases, underlined)	
D42A_F	5'-TTTGCTTATATTATTGCTTTAATCGTG-3' (Mutagenic base, underlined)	for the codon change of No. 42 residue from GAT for Asp to GCT for Ala
D42A_R	5'-GCAATAATATAAGCAAAAAAGCGTGGA-3' (Mutagenic base, underlined)	
R124A_F	5'-GGACAAATTATCTTTGCAGAGTTTGTG-3' (Mutagenic bases, underlined)	for the codon change of No. 124 residue from CGA for Arg to GCA for Ala
R124A_R	5'-GCAAAGATAATTTGTCCTTTCGTTA-3' (Mutagenic bases, underlined)	
R124K_F	5'-GGACAAATTATCTTTAAAGAGTTTGTG-3' (Mutagenic base, underlined)	for the codon change of No. 124 residue from CGA for Arg to AAA for Lys
R124K_R	5'-TTAAAGATAATTTGTCCTTTCGTTAAG-3' (Mutagenic base, underlined)	
R129A_F	5'-AGAGAGTTTGTGGGAGCTTATATAAGT-3' (Mutagenic bases, underlined)	for the codon change of No. 129 residue from CGT for Arg to

R129A_R	5'-GCTCCCACAAACTCTCTAAAGATAATT-3' (Mutagenic bases, underlined)	GCT for Ala
R129K_F	5'-AGAGAGTTTGTGGGAAAATATATAAGT-3'(Mutagenic base, underlined)	for the codon change of No. 129 residue from CGT for Arg to
R129K_R	5'-TTTTCCCACAAACTCTCTAAAGATAAT-3'(Mutagenic base, underlined)	AAA for Lys
D154A_F	5'-CATCAAGGGATCCACGCCCTATTTGCC-3' (Mutagenic base, underlined)	for the codon change of No. 154 residue from GAC for Asp to
D154A_R	5'-GCGTGGATCCCTTGATGCTTTCTCGTG-3' (Mutagenic base, underlined)	GCC for Ala
D154E_F	5'-ATCAAGGGATCCACGAACTATTTGCCG-3' (Mutagenic base, underlined)	for the codon change of No. 154 residue from GAC for Asp to
D154E_R	5'-TTCGTGGATCCCTTGATGCTTTCTCGT-3' (Mutagenic base, underlined)	GAA for Glu
D158A_F	5'-CACGACCTATTTGCCGCTACTTCTGTA-3' (Mutagenic base, underlined)	for the codon change of No. 158 residue from GAT for Asp to
D158A_R	5'-GCGGCAAATAGGTCGTGGATCCCTTG-3' (Mutagenic base, underlined)	GCT for Ala
D158E_F	5'-CGACCTATTTGCCGAGACTTCTGTAAT-3' (Mutagenic base, underlined)	for the codon change of No. 158 residue from GAT for Asp to
D158E_R	5'-CTCGGCAAATAGGTCGTGGATCCCTTG-3' (Mutagenic base, underlined)	GAG for Glu
